# Sleep Matters for Intimacy: Impact of Sleep Quality and Psychosocial Context on Female Sexual Function During Pregnancy

**DOI:** 10.3390/medicina62010150

**Published:** 2026-01-12

**Authors:** Razvan-Ionut Daniluc, Iulia Georgiana Bogdan, Alina Tischer, Marius Craina, Loredana Gabriela Stana

**Affiliations:** 1 Doctoral School, Faculty of Medicine, “Victor Babes” University of Medicine and Pharmacy Timisoara, 300041 Timisoara, Romania; razvan.daniluc@umft.ro; 2 Department of Infectious Diseases, “Victor Babes” University of Medicine and Pharmacy Timisoara, 300041 Timisoara, Romania; iulia-georgiana.bogdan@umft.ro; 3 Ear-Nose-Throat Department, “Victor Babes” University of Medicine and Pharmacy Timisoara, 300041 Timisoara, Romania; 4 Department of Obstetrics and Gynecology, Faculty of Medicine, “Victor Babes” University of Medicine and Pharmacy Timisoara, 300041 Timisoara, Romania; craina.marius@umft.ro; 5 Department I, Discipline of Anatomy and Embryology, “Victor Babes” University of Medicine and Pharmacy Timisoara, 300041 Timisoara, Romania; loredana.stana@umft.ro

**Keywords:** pregnancy, sleep–wake disorders, female sexual function, physical activity, social support, depressive symptoms, body image

## Abstract

*Background and Objectives*: Sleep disruption and reduced physical activity are common in pregnancy and may impair sexual function through mood, body-image, and relational pathways. We prospectively examined whether sleep quality and physical activity predicted third-trimester sexual function in a Romanian antenatal cohort, and explored psychosocial correlates. *Materials and Methods:* In a single-center cohort, 102 pregnant adults were enrolled ≤ 20 weeks and followed to the third trimester. Sleep (Pittsburgh Sleep Quality Index, PSQI), sexual function (Female Sexual Function Index–Romanian version, FSFI-RO), physical activity (IPAQ-SF), depressive symptoms (PHQ-9), body-image avoidance (Body Exposure during Sexual Activities Questionnaire, BESAQ), and perceived social support (MSPSS) were assessed. Groups were defined by mid-/late-pregnancy sleep (good, PSQI ≤ 5; poor, PSQI > 5). Analyses used *t*-tests, Pearson correlations, multivariable linear regression for FSFI-Total, and logistic regression for FSFI-Total < 26.55. *Results*: Compared with good sleepers (n = 56), women with poor sleep (n = 46) had lower third-trimester FSFI-Total (24.4 ± 3.9 vs. 27.9 ± 4.3; *p* < 0.001) and higher odds of FSFI-defined dysfunction (adjusted OR 121.1; 95% CI 19.2–763.0; *p* < 0.001). FSFI-Total correlated with worse sleep (PSQI r = −0.42), depressive symptoms (PHQ-9 r = −0.36), social support (MSPSS r = 0.40), body-image avoidance (BESAQ r = −0.34) and physical activity (IPAQ-SF r = 0.24; all *p* ≤ 0.015). In adjusted models (R^2^ = 0.42), higher MSPSS (β = 0.26; *p* = 0.004) was protective, whereas PSQI (β = −0.24; *p* = 0.008), ΔPHQ-9 (β = −0.19; *p* = 0.023), BESAQ (β = −0.17; *p* = 0.031), and higher BMI (β = −0.14; *p* = 0.049) predicted lower FSFI-Total. *Conclusions*: In this antenatal cohort, poor sleep was strongly and independently associated with lower sexual function, with meaningful contributions from social support, mood, body-image cognition, and physical activity, highlighting sleep as a clinically actionable target for preserving sexual well-being in pregnancy.

## 1. Introduction

Sleep disruption is highly prevalent across pregnancy and has been linked to broader adverse physiological sequelae. Meta-analytic evidence shows that up to half of pregnant individuals report clinically meaningful poor sleep quality on the Pittsburgh Sleep Quality Index (PSQI) and that sleep complaints escalate with advancing gestation [1]. Short or fragmented sleep is also associated with cardiometabolic risks relevant to obstetric care—gestational diabetes, hypertensive disorders, and adverse birth outcomes—highlighting sleep as a plausible upstream driver of fatigue, mood disturbance, altered nociception, and reduced sexual responsiveness [2]. Beyond pregnancy, population data associate poorer sleep with higher odds of global sexual dysfunction and decrements across desire, lubrication, orgasm, satisfaction, and pain domains, underscoring bidirectional sleep–sexual health links [3]. Pregnancy-specific data further suggest that insomnia severity and sexuality are intertwined, even when PSQI totals and sexual function do not correlate linearly [4].

The Pittsburgh Sleep Quality Index remains a brief, widely used measure of global sleep quality and has been applied in Romanian adult cohorts using Romanian-language versions [5]. In parallel, physical activity (PA) is a modifiable behavior that improves sleep consolidation, reduces depressive symptoms, and supports weight management and vitality; the short-form International Physical Activity Questionnaire (IPAQ-SF) offers a cost-effective surveillance tool and has been used in Romanian student and adult samples [6,7].

Female sexual function is multidimensional—desire, arousal, lubrication, orgasm, satisfaction, pain—and the Romanian version of the Female Sexual Function Index (FSFI-RO) shows strong psychometrics, supporting reliable assessment in Romanian-speaking populations [8]. Body-image-related avoidance during sexual activity, indexed by the Body Exposure during Sexual Activities Questionnaire (BESAQ), can constrain intimacy and predicts poorer sexual function; this construct has also been operationalized in Romanian cohorts studying sexuality during pregnancy and reproductive health [9,10,11]. These instruments together allow simultaneous capture of function and a key interpersonal-cognitive constraint (body exposure/avoidance), which may mediate or modify sleep/PA effects on sexual well-being.

Mood is a proximal determinant of sexual health in pregnancy. The Patient Health Questionnaire-9 (PHQ-9) is free, brief, and robust across settings, facilitating standardized quantification of depressive symptoms that often co-occur with sleep complaints and diminished sexual function [12,13]. Perceived social support aligns with partner-level buffering effects on stress and sexual well-being; Romanian-language validations of the Multidimensional Scale of Perceived Social Support (MSPSS) provide culturally appropriate measurement for this contextual resource [14,15].

Against this backdrop, a focus on modifiable, clinically actionable levers is compelling. Sleep quality and PA can change rapidly with brief behavioral counseling, and both plausibly influence sexual function via mood and body-image pathways during pregnancy [2,4,7,9,11,12]. Leveraging a consistent sexual function framework (FSFI-RO) while integrating PSQI, IPAQ-SF, PHQ-9, MSPSS, and BESAQ enables a novel comparison—good vs. poor sleep quality—with PA level as a contextual modifier and mechanistic attention to depressive symptoms, body-image avoidance, and social support. We therefore hypothesized that (i) good sleep would be associated with higher third-trimester FSFI-Total; (ii) PA would additively improve FSFI; and (iii) depressive symptoms and body-image avoidance would partially mediate these associations, with social support exerting an independent positive effect.

## 2. Materials and Methods

### 2.1. Setting, Participants, and Ethics

Consecutive pregnant adults receiving routine antenatal care at the Obstetrics & Gynecology Department, “Victor Babeș” University of Medicine and Pharmacy, Timișoara, were enrolled at ≤20 gestational weeks (T1, baseline). Follow-ups occurred mid-gestation (T2, 20–27 + 6 weeks) and in the third trimester (T3, ≥28 weeks). Inclusion criteria were age ≥ 18 years, singleton viable pregnancy, and Romanian fluency for self-report instruments. Exclusion criteria were active severe psychiatric illness requiring treatment escalation, major fetal anomaly at enrollment, activity-restricting obstetric complications at enrollment (threatened preterm labor), documented cognitive impairment, or planned relocation before delivery. Written informed consent was obtained at T1; the protocol was approved by the institutional ethics committee and adhered to the Declaration of Helsinki.

Recruitment occurred between March 2024 and October 2025. Consecutive eligible patients attending routine antenatal visits were approached in clinic by study staff. Of 156 patients assessed for eligibility, 28 declined participation and 20 were excluded (most commonly due to insufficient Romanian fluency for self-report instruments (n = 7), activity-restricting obstetric complications at enrollment such as threatened preterm labor (n = 6), planned relocation or inability to complete follow-up before delivery (n = 4), and active severe psychiatric illness requiring treatment escalation (n = 3). A total of 108 participants provided baseline data (T1), and 102 had complete third-trimester FSFI outcome data and were included in the analytic cohort.

PICO statement: Population: pregnant adults receiving antenatal care at a tertiary center in Timișoara (Romania). Intervention/Exposure: better sleep quality (PSQI ≤ 5) and higher physical activity (IPAQ-SF moderate/high). Comparator: poorer sleep (PSQI > 5) and/or low physical activity. Outcomes: primary—third-trimester FSFI-Total; secondary—domain-specific FSFI, trimester trajectories, and associations with PHQ-9, MSPSS, BESAQ, and IPAQ-SF; exploratory—proportion below the non-pregnancy FSFI cutoff (26.55). Design: prospective observational cohort.

### 2.2. Measures and Instruments

The Female Sexual Function Index (FSFI) is a 19-item self-report measure covering six domains—desire (items 1–2), arousal (3–6), lubrication (7–10), orgasm (11–13), satisfaction (14–16), and pain (17–19). Items are summed within domains and multiplied by established factors (desire 0.6; arousal 0.3; lubrication 0.3; orgasm 0.4; satisfaction 0.4; pain 0.4) to yield domain scores (0–6) and a total score (2–36), where higher indicates better function. A total score ≤ 26.55 has been proposed to screen for global dysfunction in non-pregnant populations. Because pregnancy-specific diagnostic cutoffs are not uniformly established, this threshold was used only for exploratory, descriptive reporting and a secondary logistic model; primary inference was based on continuous FSFI outcomes [16].

The Body Exposure during Sexual Activities Questionnaire (BESAQ) assesses situational appearance-focused self-consciousness during intimacy. The 28 items are rated 0–4; the recommended score is the mean across items (0–4), with higher indicating greater avoidance/monitoring. BESAQ shows good construct validity and has been adapted/validated across populations, including women of reproductive age and pregnant cohorts [17].

The Pittsburgh Sleep Quality Index (PSQI) comprises seven components (subjective quality, latency, duration, efficiency, disturbances, medication use, daytime dysfunction), each scored 0–3; the components sum to a global score of 0–21, with >5 indicating poor sleep (diagnostic sensitivity ~90%, specificity ~86% in original validation). A Romanian translation is available and has been used in Romanian adult cohorts [18].

The Patient Health Questionnaire-9 (PHQ-9) scores each symptom 0–3 for a total of 0–27; conventional cut-points 5/10/15/20 indicate mild/moderate/moderately severe/severe severity. Psychometric validation supports its use [12].

The 12-item Multidimensional Scale of Perceived Social Support (MSPSS) yields subscale means (Family, Friends, Significant Other) and a total mean, each ranging 1–7, where higher reflects greater perceived support. A Romanian version has demonstrated good reliability and factorial validity [19].

The International Physical Activity Questionnaire—Short Form (IPAQ-SF; 7-day recall) computes MET-minutes/week using standard MET values (walking 3.3, moderate 4.0, vigorous 8.0 METs) and classifies respondents as Low, Moderate, or High per the 2005 scoring guidelines. For analysis, Moderate and High were combined as “moderate/high,” contrasted with “low”. The IPAQ-SF is widely used in Romania, including university and adult samples [20,21].

Pre-specified covariates were age, first-trimester BMI, residence (urban/rural), education, marital status, smoking, prior abortion, menstrual-cycle regularity, and trimester-specific PHQ-9, MSPSS, BESAQ, PSQI, and IPAQ-SF indices (as detailed above).

### 2.3. Procedures and Timing

At T1 (≤20 weeks) participants completed sociodemographics, FSFI-RO, BESAQ, and PHQ-9; at T2, PSQI, IPAQ-SF, and PHQ-9 were collected; at T3, FSFI-RO, BESAQ, MSPSS, and PSQI were repeated. Surveys were self-completed without partners present to minimize social desirability bias; staff verified completeness at point of collection and clarified missing/skipped items immediately. Romanian-language instruments were used in all cases, drawing on validated translations where available (FSFI-RO, PHQ-9-RO, MSPSS-RO, PSQI-RO). Sleep quality and physical activity were assessed before the third-trimester FSFI endpoint, whereas social support and body-image avoidance were measured at the same third-trimester timepoint as FSFI.

### 2.4. Study Groups, Outcomes, and Hypotheses

Primary exposure groups were defined a priori by mid-gestation sleep quality (T2 PSQI): good sleep (PSQI ≤ 5) versus poor sleep (PSQI > 5). PA served as a contextual modifier, creating a 2 × 2 stratification (sleep × PA). The primary outcome was third-trimester FSFI-Total. Secondary outcomes included FSFI domain scores at T3, FSFI trajectories across trimesters, and cross-sectional associations between T3 FSFI-Total and PSQI, PHQ-9, MSPSS, BESAQ, and IPAQ-SF. Exploratory descriptive analyses reported proportions below FSFI-Total 26.55, recognizing this screening threshold was derived outside pregnancy.

### 2.5. Statistical Analysis

Analyses were performed using SPSS (version v.27). Continuous variables are presented as mean ± SD; normality was assessed via Shapiro–Wilk and Q–Q plots. Between-group comparisons used Welch’s *t*-tests (continuous) and χ^2^ or Fisher’s exact tests (categorical). FSFI domain *p*-values were adjusted with Benjamini–Hochberg at FDR 0.05. Correlations between T3 FSFI-Total and psychosocial/sleep/PA indices used Pearson’s r (Spearman’s ρ in sensitivity analyses for non-normal pairs). The primary multivariable model was linear regression predicting T3 FSFI-Total with pre-specified covariates (age, BMI, rural residence, IPAQ category, PSQI, BESAQ, ΔPHQ-9, MSPSS). ΔPHQ-9 (T1 → T2) was prespecified to capture early symptom trajectory, which may precede later sexual-function changes and reduce overlap with third-trimester outcome measurement. Predictors measured concurrently with the outcome were interpreted as correlates rather than temporally antecedent risk factors. Model diagnostics evaluated linearity (component-plus-residual plots), homoscedasticity (Breusch–Pagan; HC3 robust SEs in sensitivity), normality of residuals, leverage (hat values), and multicollinearity (VIF < 5). The prespecified subgroup analysis contrasted the four sleep × PA cells using Welch ANOVA. Two-sided α = 0.05.

Model diagnostics included inspection of residual plots, assessment of influential observations, and collinearity evaluation using variance inflation factors (all VIFs < 2.30; range 1.12–2.30). Because this was a prospective observational cohort, recruitment was planned pragmatically; we targeted approximately 100 participants to support multivariable modeling with prespecified covariates while maintaining an acceptable events-per-parameter ratio for secondary logistic analyses.

## 3. Results

Groups were balanced on age, residence, education, marital status, and smoking (all *p* > 0.20), but women with poor sleep entered pregnancy with a higher BMI (24.6 ± 3.3 vs. 23.2 ± 3.0 kg/m^2^; *p* = 0.029) and markedly worse psychosocial/somatic profiles: lower perceived social support (MSPSS 4.7 ± 1.0 vs. 5.6 ± 0.8; *p* < 0.001), more depressive symptoms at baseline (PHQ-9 7.1 ± 3.5 vs. 4.3 ± 3.0; *p* < 0.001), and lower physical activity at mid-gestation (IPAQ-SF 1390.0 ± 650.0 vs. 1860.0 ± 730.0 MET-min/week; *p* = 0.001). Sleep quality separation was robust (PSQI 8.7 ± 1.3 vs. 4.1 ± 0.9; *p* < 0.001), and body-image-related avoidance was higher in the poor-sleep group (BESAQ 2.2 ± 0.6 vs. 1.6 ± 0.5; *p* < 0.001) (Table 1).

FSFI-Total scores were consistently higher among those with good sleep at every timepoint, with gaps of 2.9 points in T1 (29.3 ± 4.5 vs. 26.4 ± 4.2; *p* = 0.001), 3.4 points in T2 (28.6 ± 4.4 vs. 25.2 ± 4.1; *p* < 0.001), and 3.5 points at the primary T3 endpoint (27.9 ± 4.3 vs. 24.4 ± 3.9; *p* < 0.001). Both groups exhibited modest trimester-related declines, but the between-group separation remained stable, underscoring sleep quality as a persistent correlate of global sexual function in pregnancy (Table 2).

At T3, good sleep was associated with broadly better domain scores: desire (4.4 ± 0.8 vs. 3.8 ± 0.9; *p* = 0.002), arousal (4.5 ± 0.9 vs. 4.0 ± 0.9; *p* = 0.0076), lubrication (4.9 ± 0.8 vs. 4.2 ± 0.9; *p* = 0.0005), orgasm (4.6 ± 0.9 vs. 4.0 ± 0.9; *p* = 0.0017), and satisfaction (4.8 ± 0.8 vs. 4.2 ± 0.9; *p* = 0.0013). The largest absolute separations were observed for lubrication (+0.7) and satisfaction (+0.6). Pain showed a smaller, non-significant difference (4.5 ± 1.0 vs. 4.2 ± 1.0; *p* = 0.135), suggesting sleep quality maps most strongly onto desire-arousal-lubrication-orgasm-satisfaction pathways rather than dyspareunia (Table 3).

**Figure 1:**
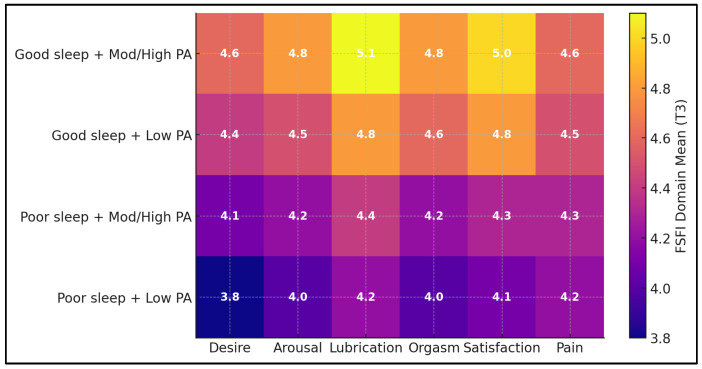
Female Sexual Function Index domain profiles by sleep quality and physical-activity subgroup (heatmap). Domain means are shown for desire, arousal, lubrication, orgasm, satisfaction, and pain, stratified by sleep quality (good vs. poor) and physical activity (low vs. moderate/high). The color scale reflects lower-to-higher domain scores (0–6). Cell labels display the domain mean (one decimal). Abbreviations: FSFI, Female Sexual Function Index; PA, physical activity.

Correlation analyses indicated that worse sleep quality was moderately associated with lower third-trimester sexual function (*r* = −0.42, *p* < 0.001). Depressive symptoms also showed a moderate inverse association with sexual function (*r* = −0.36, *p* < 0.001). In contrast, higher perceived social support correlated positively with sexual function (*r* = 0.40, *p* < 0.001). Body-image avoidance during intimacy was inversely associated with sexual function (*r* = −0.34, *p* < 0.001). Physical activity showed a smaller positive association (*r* = 0.24, *p* = 0.015), consistent with a more modest relationship than sleep and psychosocial factors (Table 4 and Figure 1).

In the adjusted model (R^2^ = 0.42), higher perceived social support (MSPSS) independently predicted better sexual function (β = 0.26; 95% CI 0.08–0.44; *p* = 0.004), while worse sleep (PSQI; β = −0.24; −0.42 to −0.06; *p* = 0.008), worsening depressive symptoms (ΔPHQ-9; β = −0.19; −0.35 to −0.03; *p* = 0.023), and greater body-image avoidance (BESAQ; β = −0.17; −0.32 to −0.02; *p* = 0.031) were detrimental. Higher early-pregnancy BMI showed a small negative association (β = −0.14; −0.29 to −0.00; *p* = 0.049). Physical activity trended positive (β = 0.12; *p* = 0.089), whereas rural residence and age were null (both *p* > 0.25), as presented in Table 5

A clear stepwise gradient emerged across the four cells (Welch ANOVA F(3,98) = 8.50; *p* < 0.001): Good sleep + Moderate/High PA had the highest FSFI-Total (28.9 ± 3.8), followed by Good sleep + Low PA (27.1 ± 4.3), Poor sleep + Moderate/High PA (25.5 ± 3.6), and Poor sleep + Low PA (23.7 ± 3.9). Key pairwise differences favored good-sleep groups over their poor-sleep counterparts (Good + Mod/High vs. Poor + Mod/High *p* = 0.0119; Good + Low vs. Poor + Low *p* = 0.0073), whereas within-sleep PA contrasts did not survive BH adjustment (both *p* > 0.11), reinforcing sleep quality as the principal driver (Table 6).

**Figure 2:**
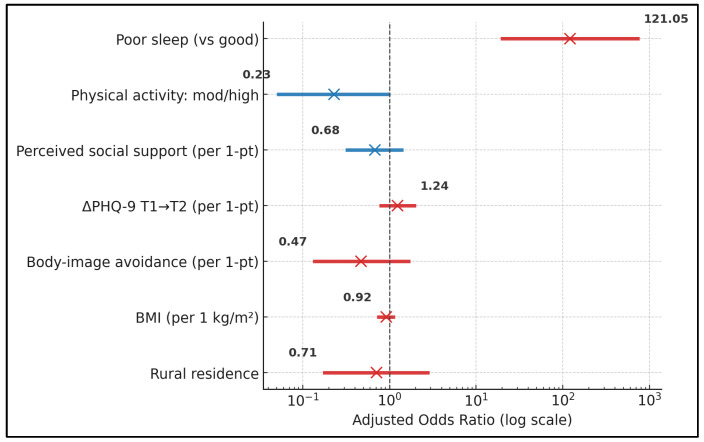
Multivariable association between sleep quality and third-trimester Female Sexual Function Index-defined dysfunction (forest plot). Adjusted odds ratios (points) and 95% confidence intervals (lines) are shown for predictors included in the logistic regression model. Odds ratios are displayed on a logarithmic scale. The sleep variable contrasts poor vs. good sleep. Abbreviations: PHQ-9, Patient Health Questionnaire-9; BMI, body mass index.

Because the threshold is derived from non-pregnant samples, dysfunction estimates based on FSFI < 26.55 should be interpreted as comparative rather than diagnostic. Poor sleep conferred a higher adjusted likelihood of FSFI-defined dysfunction (FSFI-Total < 26.55) with an odds ratio of 121.05 (95% CI 19.21–763.01; *p* < 0.001), dwarfing effects of other covariates. Moderate/high physical activity showed a borderline protective association (0.23; 0.05–1.03; *p* = 0.055). Estimates for MSPSS, ΔPHQ-9, BESAQ, BMI, and rural residence were imprecise with CIs spanning unity (all *p* ≥ 0.26), indicating that, in categorical risk terms, sleep quality dominated the prediction of dysfunction at T3 (Table 7 and Figure 2). The very large odds ratio for poor sleep may reflect sparse-data effects; therefore, categorical dysfunction findings are interpreted cautiously and are supported primarily by continuous-outcome analyses. These subgroup differences are visualized in Figure 1, which illustrates consistently higher domain scores in the good-sleep groups across desire, arousal, lubrication, orgasm, and satisfaction. Predictors of FSFI-defined dysfunction from the adjusted logistic regression are summarized in Figure 2.

## 4. Discussion

### 4.1. Analysis of Findings

The present cohort showed a persistent separation in global and domain-specific sexual function across pregnancy between women with good vs. poor sleep, with the largest gaps in lubrication and satisfaction and a smaller, non-significant difference for pain. These patterns converged with multi-setting reports that FSFI scores decline as gestation advances and that the most pronounced decrements cluster in desire–arousal–lubrication–orgasm domains rather than dyspareunia, although domain-level results are heterogeneous across populations [22,23,24,25]. Large prospective and cross-sectional series likewise documented trimester-related reductions in sexual activity and greater odds of FSFI-defined dysfunction by the third trimester, reinforcing that the magnitude and direction of our domain effects align with established trajectories in pregnancy samples [24]. Although the pattern is consistent with a potential pathway linking sleep, mood, and sexual function, formal mediation modeling was not performed in this cohort and should be evaluated in larger samples with clear temporal separation of mediator and outcome.

Interpretation of PSQI findings in pregnancy should consider that the PSQI may behave as a multidimensional measure in perinatal samples, and some sleep complaints typical of pregnancy may not map uniformly onto its components. Prior perinatal psychometric studies suggest that insomnia-focused instruments can complement global PSQI scoring when the aim is to capture nocturnal symptom burden most closely linked to daytime fatigue and sexual responsiveness. Accordingly, future work may benefit from combining global sleep quality indices with insomnia-specific measures [26,27,28,29,30,31,32,33,34].

The correlation pattern supports a coherent biopsychosocial framework in which sleep quality and mood relate to sexual function alongside interpersonal resources and body-image cognitions. The magnitude of associations was moderate for sleep and social support and somewhat smaller for body-image avoidance and physical activity, suggesting that while activity may contribute, sleep and relational/psychological context account for a larger proportion of variability in sexual function during late pregnancy.

The Sleep × Physical-Activity (PA) gradient observed herein—highest FSFI totals among women with good sleep and moderate/high PA—was directionally consistent with literature linking antenatal PA to better sleep consolidation and overall well-being. A recent meta-analysis concluded that PA reduces sleep disorders during pregnancy, and an observational study associated moderate-intensity activity (≥7.5 MET-h/week) and better PSQI scores with improved mental health and lower adverse-outcome risk, suggesting a plausible pathway whereby PA indirectly supports sexual function via better sleep and mood. Although our within-sleep PA contrasts were attenuated after false-discovery control—likely reflecting limited power—interventional evidence (e.g., a yoga program improving FSFI domains in pregnant participants) supports the biological plausibility that movement-based approaches can augment sexual well-being during gestation [29,30,31].

Psychosocial context also mattered. Higher perceived social support independently predicted better sexual function, in line with reports that spousal/partner support correlates with higher FSFI scores in primigravid women and may buffer the impact of stressors on intimacy. In parallel, our data linked worsening depressive symptoms with lower FSFI; this accords with evidence that poor antenatal sleep quality increases depressive symptoms and that sleep disturbance is a risk factor for perinatal mood morbidity, providing a coherent explanatory pathway (sleep → mood → sexual function) that complements our regression and correlation results [30,32,33].

Body-image-related avoidance was inversely associated with sexual function in the third trimester, echoing studies that connect negative body perception with reduced desire, arousal, and satisfaction during late pregnancy. Importantly, contemporary work suggests that the body-image/sexual function link may be mediated by cognitive distraction and sexual distress rather than operating as a simple direct effect, and that body-image dissatisfaction during pregnancy is shaped by modifiable factors—including sleep quality, PA, and mood—that were salient in our cohort. These findings support integrated counseling that addresses sleep, mood, and body-image concerns alongside sexual well-being [35,36,37].

Recent longitudinal and clinical literature further highlights the heterogeneity of perinatal mood trajectories and identifies early postpartum and prepartum affective vulnerabilities as clinically relevant risk markers, supporting integrated sleep–mood screening approaches in obstetric care [38,39].

From a clinical perspective, antenatal sleep screening can be implemented using brief questionnaires, followed by stepped care. Low-intensity interventions may include sleep-hygiene counseling (regular sleep schedule, stimulus control, limiting evening screen exposure, and managing nocturnal discomfort). For persistent insomnia symptoms, referral pathways for cognitive behavioral therapy for insomnia (CBT-I) or structured behavioral programs could be considered where available. Integrating mood screening and brief supportive counseling may further improve sleep-related distress and support sexual well-being.

Sociocultural norms regarding sexuality in pregnancy may influence disclosure and interpretation of questionnaire-based sexual-function measures. In Romania, attitudes toward sexual activity during pregnancy can vary across generations, educational levels, and urban–rural contexts, potentially affecting self-report via privacy concerns or perceived stigma. These factors may limit generalizability and underscore the importance of culturally sensitive counseling and confidential assessment.

Finally, the small adverse association between early-pregnancy BMI and FSFI-Total in adjusted analyses should be interpreted cautiously. While several pregnancy cohorts describe global FSFI declines across trimesters, the contribution of adiposity per se appears inconsistent and may be confounded by co-occurring sleep fragmentation, weight-related body-image concerns, and lubrication/arousal changes. Large observational series—particularly those parsing domain-level outcomes—suggest that relationship quality and partner factors often outweigh anthropometry in explaining variance in sexual function during pregnancy, which is compatible with our modest BMI effect and the strong role of social support observed here [23,40].

### 4.2. Study Limitations

The observational, single-center design limits causal inference and generalizability beyond Romanian tertiary care. All key constructs (sleep, sexual function, mood, PA, social support, body-image avoidance) were self-reported, introducing potential reporting and common-method biases; objective actigraphy or device-based PA was not collected. Sexual function was analyzed continuously, and the <26.55 FSFI threshold (derived in non-pregnant cohorts) was used only descriptively, yet misclassification is still possible. Residual confounding (relationship factors, partner sexual health, obstetric comorbidities, medication use) cannot be excluded. Although sleep quality was assessed before the primary third-trimester sexual-function endpoint, several psychosocial covariates (social support and body-image avoidance) were measured contemporaneously with the outcome, limiting causal interpretation of these associations. Moreover, false-discovery control was applied for domain comparisons, multiple testing may still inflate type-I error. The sample size, while adequate for primary analyses, constrained precision for some covariates and interaction tests. We did not assess relationship quality, partner sexual health, or frequency of sexual activity, which are key determinants of FSFI scores and may confound observed associations. Future studies should incorporate validated dyadic measures (relationship satisfaction/communication, partner support) and behavioral indices (sexual activity frequency and distress) to better separate sleep-related changes from relationship-context effects.

## 5. Conclusions

In this cohort, sleep quality emerged as a strong, clinically actionable correlate of third-trimester sexual function, complemented by the beneficial influences of greater perceived social support, lower depressive symptom burden, healthier body-image cognitions, and modestly higher physical activity. Antenatal care might incorporate brief sleep screening (PSQI), targeted behavioral sleep counseling, and integrated psychosocial support (mood management, body-image-informed counseling, partner/peer support). Future multicenter studies using objective sleep/PA measures and interventional designs are warranted to test whether improving sleep and psychosocial context yields durable gains in sexual well-being during pregnancy.

## Figures and Tables

**Table 1:** Baseline characteristics by mid-gestation sleep quality (T2 PSQI ≤ 5 vs. >5).

Characteristic	Good Sleep (PSQI ≤ 5; n = 56)	Poor Sleep (PSQI > 5; n = 46)	*p*-Value
Age, years	28.7 ± 4.9	28.1 ± 5.1	0.549
BMI, kg/m^2^ (T1)	23.2 ± 3.0	24.6 ± 3.3	0.029
Rural residence, n	18	20	0.215
Higher education, n	24	17	0.489
Unmarried, n	11	12	0.286
Current smoker, n	8	9	0.332
MSPSS (1–7)	5.6 ± 0.8	4.7 ± 1.0	<0.001
PHQ-9 (T1)	4.3 ± 3.0	7.1 ± 3.5	<0.001
IPAQ-SF MET-min/week (T2)	1860.0 ± 730.0	1390.0 ± 650.0	0.001
PSQI global (T2)	4.1 ± 0.9	8.7 ± 1.3	<0.001
BESAQ (T1; 0–4)	1.6 ± 0.5	2.2 ± 0.6	<0.001

Values are mean ± standard deviation or count. Group comparisons used Welch’s *t*-test for continuous variables and χ^2^ or Fisher’s exact test for categorical variables. PSQI, Pittsburgh Sleep Quality Index; BMI, body mass index; PHQ-9, Patient Health Questionnaire-9; MSPSS, Multidimensional Scale of Perceived Social Support; IPAQ-SF, International Physical Activity Questionnaire—Short Form; MET, metabolic equivalent; BESAQ, Body Exposure during Sexual Activities Questionnaire.

**Table 2:** FSFI-Total across trimesters by sleep quality.

Timepoint	Good Sleep (n = 56)	Poor Sleep (n = 46)	*p*-Value
Trimester 1	29.3 ± 4.5	26.4 ± 4.2	0.001
Trimester 2	28.6 ± 4.4	25.2 ± 4.1	<0.001
Trimester 3 (primary)	27.9 ± 4.3	24.4 ± 3.9	<0.001

Test: Welch *t*-test; FSFI, Female Sexual Function Index.

**Table 3:** FSFI domains at third trimester.

Domain (T3)	Good Sleep (n = 56)	Poor Sleep (n = 46)	*p* (Adjusted)
Desire	4.4 ± 0.8	3.8 ± 0.9	0.002
Arousal	4.5 ± 0.9	4.0 ± 0.9	0.0076
Lubrication	4.9 ± 0.8	4.2 ± 0.9	0.0005
Orgasm	4.6 ± 0.9	4.0 ± 0.9	0.0017
Satisfaction	4.8 ± 0.8	4.2 ± 0.9	0.0013
Pain	4.5 ± 1.0	4.2 ± 1.0	0.135

Test: Welch *t*-test; FSFI, Female Sexual Function Index; T3, third trimester.

**Table 4:** Correlations between third-trimester Female Sexual Function Index total score and sleep/psychosocial variables.

Predictor	Pearson r	*p*-Value
PSQI global (higher = worse sleep)	−0.42	<0.001
PHQ-9 (T2)	−0.36	<0.001
MSPSS (T3)	0.4	<0.001
BESAQ (T3)	−0.34	<0.001
IPAQ-SF MET-min/week (T2)	0.24	0.015

Pearson correlation coefficients are shown. PSQI, Pittsburgh Sleep Quality Index; PHQ-9, Patient Health Questionnaire-9; MSPSS, Multidimensional Scale of Perceived Social Support; BESAQ, Body Exposure during Sexual Activities Questionnaire; IPAQ-SF, International Physical Activity Questionnaire—Short Form; T2, second trimester; T3, third trimester.

**Table 5:** Multivariable predictors of third-trimester FSFI-Total (linear regression).

Predictor	β (Standardized)	95% CI	*p*-Value
MSPSS (T3)	0.26	0.08 to 0.44	0.004
PSQI global (T2)	−0.24	−0.42 to −0.06	0.008
ΔPHQ-9 (T1 → T2)	−0.19	−0.35 to −0.03	0.023
BESAQ (T3)	−0.17	−0.32 to −0.02	0.031
BMI (T1), kg/m^2^	−0.14	−0.29 to −0.00	0.049
IPAQ-SF (T2)	0.12	−0.02 to 0.26	0.089
Rural residence	−0.08	−0.23 to 0.06	0.252
Age, years	−0.05	−0.19 to 0.09	0.511

Model: R^2^ = 0.42; diagnostics acceptable; β, standardized regression coefficient; CI, confidence interval; MSPSS, Multidimensional Scale of Perceived Social Support; PSQI, Pittsburgh Sleep Quality Index; ΔPHQ-9, change in PHQ-9 from T1 to T2; BESAQ, Body Exposure during Sexual Activities Questionnaire; BMI, body mass index; IPAQ-SF, International Physical Activity Questionnaire—Short Form; T3, third trimester.

**Table 6:** Sleep × Physical Activity subgroup analysis of FSFI-Total (T3).

Group (n)	FSFI-Total (Mean ± SD)
Good sleep + Moderate/High PA (n = 28)	28.9 ± 3.8
Good sleep + Low PA (n = 28)	27.1 ± 4.3
Poor sleep + Moderate/High PA (n = 20)	25.5 ± 3.6
Poor sleep + Low PA (n = 26)	23.7 ± 3.9

Overall Welch ANOVA: F(3,98) = 8.50; *p* < 0.001. Key pairwise Welch t (BH-adjusted): Good + Mod/High vs. Poor + Mod/High *p* = 0.0119; Good + Low vs. Poor + Low *p* = 0.0073; within-sleep PA contrasts were not significant after BH (both *p* > 0.11); FSFI, Female Sexual Function Index; PA, physical activity); Welch ANOVA, Welch’s analysis of variance; T3, third trimester.

**Table 7:** Adjusted odds of third-trimester sexual dysfunction (FSFI-Total < 26.55).

Predictor	Adjusted OR	95% CI	*p*-Value
Poor sleep (vs. good)	121.05	19.21–763.01	<0.001
Physical activity: moderate/high (vs. low)	0.23	0.05–1.03	0.055
Perceived social support (MSPSS, per 1-point)	0.68	0.31–1.45	0.314
ΔPHQ-9 T1 → T2 (per 1-point)	1.24	0.76–2.02	0.398
Body-image avoidance (BESAQ, per 1-point)	0.47	0.13–1.74	0.261
BMI (per 1 kg/m^2^)	0.92	0.72–1.16	0.472
Rural residence (yes vs. no)	0.71	0.17–2.90	0.631

FSFI, Female Sexual Function Index; MSPSS, Multidimensional Scale of Perceived Social Support; ΔPHQ-9, change in PHQ-9 from T1 to T2; BESAQ, Body Exposure during Sexual Activities Questionnaire; BMI, body mass index; CI, confidence interval.

## Data Availability

The data presented in this study are available on request from the corresponding author.
